# Primary intestinal Burkitt's lymphoma in a Syrian child with a challenging initial presentation: A case report and literature review

**DOI:** 10.1016/j.amsu.2022.103453

**Published:** 2022-03-03

**Authors:** Sawsan Ismail, Seif-Aldin Abdulrahman, Ibrahim Muhammad, Abdulmoniem Ghanem, Ali Daoud, Zuheir Alshehabi

**Affiliations:** a Department of Pathology, Faculty of Medicine, Cancer Research Center, Tishreen University, Lattakia, Syria; b Faculty of Medicine, Cancer Research Center, Tishreen University, Lattakia, Syria; c Department of Pediatrics, Faculty of Medicine, Tishreen University, Lattakia, Syria

**Keywords:** Burkitt's lymphoma, Non-hodgkin lymphoma, Ascites, Cytology, Histopathology

## Abstract

**Introduction:**

Burkitt's lymphoma is an aggressive type of non-Hodgkin lymphoma that represents approximately 30% of pediatric lymphomas and less than 5% of all pediatric malignancies. Although the involvement of the gastrointestinal tract is a common finding in sporadic Burkitt's lymphoma, primary intestinal lymphomas still represent a rare entity.

**Case presentation:**

We are reporting the case of an 11-year-old Syrian male who presented to our hospital with complaints of abdominal pain, distention, and tenderness. Clinical and radiologic examinations demonstrated moderate ascites with an abdominal mass. Interestingly, the cytological study of the ascites revealed the diagnosis of Burkitt's lymphoma which was later confirmed by histopathological and immunohistochemical examinations.

**Discussion:**

Pleural effusions are a common finding in extranodal lymphomas, whereas ascites is considered a rare initial presentation constituting less than 2% of lymphoma cases.

**Conclusion:**

We aimed to present an extremely rare case of a primary intestinal Burkitt's lymphoma initially presenting with ascites, highlighting the major role of the cytological study of ascites in the primary diagnosis, and the essential role of histological and immunohistochemical examinations in confirming the diagnosis in challenging cases.

## Highlights

Primary intestinal lymphomas constitute less than 2% of small intestinal malignancies.
Ascites represents an extremely rare initial presentation of non-Hodgkin lymphomas.
Cytological study of ascites represented a hard challenge in diagnosing Burkitt's lymphoma.

## List of abbreviations

**BL**: Burkitt's lymphoma
**NHL**: Non-Hodgkin lymphoma
**EBV**: Epstein-Barr virus
**HIV-1**: human immunodeficiency virus
**US**: Ultrasonography
**CT**: Computed Tomography
**PET**: Positron Emission Tomography
**CD**: Cluster of differentiation
**H&E**: Hematoxylin and Eosin
**IHC**: Immunohistochemistry
**TdT**: Terminal deoxynucleotidyl transferase
**BCL6**: B-Cell Lymphoma-6
**BCL2**: B-Cell Lymphoma-2
**WBC**: White Blood Cells
**CRP**: C-Reactive Protein
**LDH**: Lactate dehydrogenase

## 1 Introduction

Burkitt's lymphoma (BL) is an aggressive type of B-cell non-Hodgkin lymphoma (NHL) that was first defined by Dr. Dennis Burkitt in 1958 as a sarcoma of the jaw in Ugandan children [1]. BL is classified into three types that differ in epidemiological distribution, clinical presentation, and risk factors. The endemic type mostly affects facial bones and is usually associated with the Epstein-Barr virus (EBV) with high distribution among African children. The immunodeficient type is significantly associated with the human immunodeficiency virus HIV-1. The sporadic type represents approximately 30% of pediatric lymphomas and less than 5% of all pediatric malignancies. Sporadic BL mostly involves the gastrointestinal tract and is linked to EBV in less than 20% of cases [2,3]. Nevertheless, primary gastrointestinal lymphomas represent a rare entity with less than 2% of small intestinal malignancies [4]. Herein, we present the first case report from Syria -to our knowledge-of a primary intestinal Burkitt's lymphoma initially presenting with ascites which was the key to diagnosis through cytology, highlighting the importance of cytological and histological correlations for the diagnosis of challenging cases.

## 2 Case Presentation

We report the case of an 11-year-old Syrian male who presented to our hospital with complaints of intermittent generalized abdominal pain for 45 days accompanied by abdominal distention, tenderness, loss of appetite, and a rapid weight loss (15 Kgs in 6 weeks). No fever, jaundice, vomiting, or night sweats were reported. Medical, psychological, and family history were unremarkable.

**Fig. 1:**
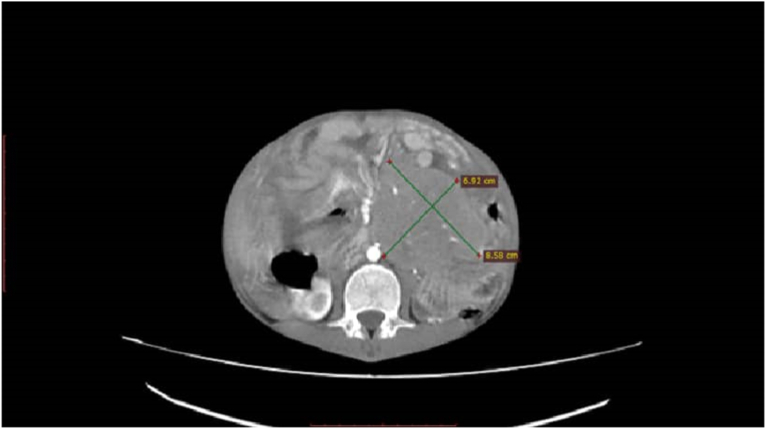
Full-body computed tomography (CT) scan demonstrating a poorly defined heterogeneous mass measuring approximately 85x70 mm.

**Fig. 2:**
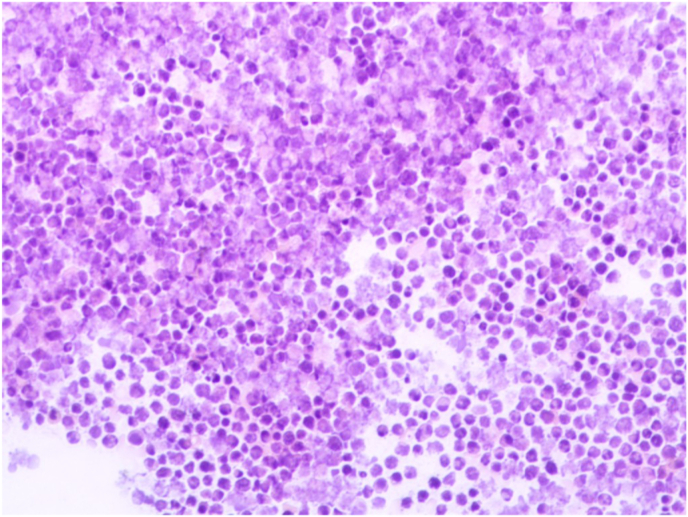
Cytological study of the ascitic fluid revealing a dense proliferation of atypical lymphocytes with basophilic vacuolated cytoplasm, round enlarged nuclei, and multiple prominent nucleoli.

**Fig. 3:**
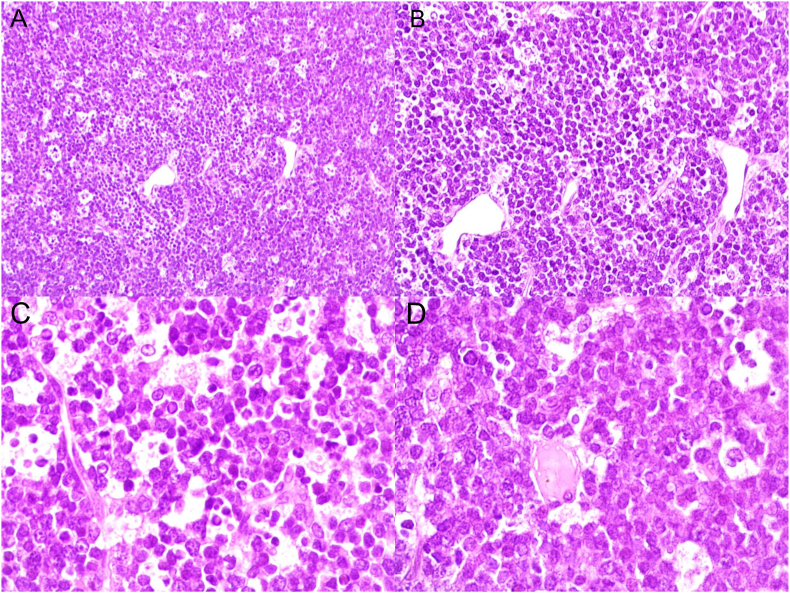
Pathological examination revealing diffuse proliferation of medium-sized monotonous atypical lymphocytes with scant basophilic cytoplasm. Numerous mitotic figures and the characteristic starry-sky appearance were present (A: Original magnification x100, B: H&E: Original magnification x200, C,D: H&E: Original magnification x400).

**Fig. 4:**
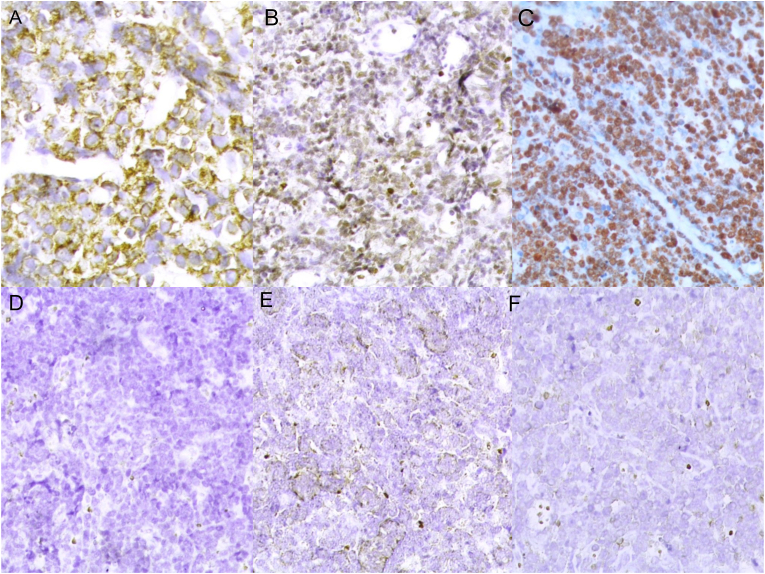
Immunohistochemical examination of the neoplasm (A: positivity for CD20. B: positivity for BCL6. C: KI-67:100%. D: Negativity for Tdt. E: negativity for BCL2. F:negativity for CD3).

Physical examination revealed mild abdominal distension and tenderness with shifting dullness. Neither hepatosplenomegaly nor lymphadenopathy was present. Laboratory examinations demonstrated WBC count 6.1 x 10^9^/L, hemoglobin value 10.8 g/dl, hematocrit value 30.3%, LDH value 4299 U/L, and CRP value 8.8 mg/L. Other tests were within normal limits. Full-body computed tomography (CT) scan demonstrated moderate ascites accompanied by a poorly defined heterogeneous mass (Fig. 1) with contrast enhancement measuring approximately (85x70)mm adhering to a thickened intestinal wall next to the ascending colon. In addition, several scattered retroperitoneal and paraaortic lymph nodes measuring up to 14 mm in diameter were observed. A preliminary diagnosis of peritoneal carcinomatosis of an undetermined origin was suspected. Nevertheless, the cytological study of the ascites revealed a dense proliferation of atypical lymphocytes with basophilic vacuolated cytoplasm, round enlarged nuclei, and multiple prominent nucleoli (Fig. 2). Primary differential diagnosis included Burkitt's lymphoma. An open incisional biopsy from the aforementioned intestinal lesion was performed, and pathological examination by two pathologists revealed diffuse proliferation of medium-sized monotonous atypical lymphocytes with scant basophilic cytoplasm. Numerous mitotic figures and the characteristic starry-sky appearance were present (Fig. 3). Immunohistochemical examination of the resected specimen revealed positivity for CD20, CD10, and BCL6, whereas CD3, TdT, and BCL2 were negative. And the proliferation index Ki-67 was approximately 100% (Fig. 4), confirming the diagnosis of Burkitt's lymphoma. Bone marrow biopsy and cerebrospinal fluid were negative for malignant cells. Molecular techniques were not available at our institution, and the diagnosis was successfully made based on detailed examinations by two pathologists (ZA and AD).

Following diagnosis, the patient was put on a chemotherapy regimen based on the LMB95 protocol developed by The Societe Francaise d’Oncologie Pediatrique (SOFP) group. Thus treatment regimen consisted of alternating courses of Cyclophosphamide, Vincristine, Prednisone, Adriamycin, and Methotrexate (COPADM), followed by consolidation courses of Cytarabine and Methotrexate (CYM). A Positron Emission Tomography/Computed Tomography (PET/CT) scan was performed six months later and revealed a complete metabolic response with no residual uptake (deauville1). The patient is currently in a good state and is being monitored regularly with CT scan to detect any recurrence. Our work was reported according to the SCARE 2020 criteria [5].

## 3 Discussion

Although the involvement of the gastrointestinal tract is a common finding in sporadic cases of Burkitt's lymphoma, primary gastrointestinal lymphomas represent a rare entity that constitutes less than 20% of extranodal non-Hodgkin lymphomas and less than 2% of small intestinal malignancies [4].

Sporadic BL usually affects pediatric populations with disparities in age distributions. In a study by Mbulaiteye S et al., sporadic BL was more predominant in younger ages (3–5) years old [2,6], whereas our case was diagnosed in an 11-year-old patient.

Pleural effusions are a common finding in extranodal lymphomas, whereas ascites is considered a rare initial presentation [7]. In a study conducted by Das et al. on serous effusions, ascites was reported in only 0.8% of NHLs, whereas pleural effusions were reported in approximately 8.6% of cases [8]. Another study by Mahmood et al. on 100 patients with malignant ascites demonstrated that lymphoma was observed as the underlying cause in only 2% of cases [9].

Among lymphoma subtypes, T-cell lymphomas were more associated with serous effusions than B-cell malignancies. Furthermore, lymphoblastic lymphoma appeared to be the most common B-cell NHL associated with serous effusions [10]. Our manuscript, however, demonstrates a rare case of Burkitt's lymphoma initially presenting with ascites.

Radiologic techniques could be helpful in the diagnosis and management of gastrointestinal neoplasms. In pediatric populations, ultrasonography (US) is preferable as a preliminary imaging technique due to the absence of ionizing radiation risk in addition to its role in evaluating peripheral lymph nodes and superficial lesions structures. Nevertheless, computed tomography (CT) scan with intravenous contrast provides a better evaluation of tumor infiltration in addition to its major role in whole-body evaluation. Furthermore, positron emission tomography (PET) scan combines anatomic detection with metabolic and functional information, which makes it a preferable method to predict treatment response [2,11,12]. In our case, a CT scan was performed in the initial diagnosis, and PET/CT scan was conducted in monitoring the patient's response to treatment.

Another interesting point in our manuscript is highlighting the role of cytology in the preliminary diagnosis. Due to the rapid proliferation of tumor cells and the risk of tumor lysis syndrome in aggressive neoplasms including BL, the cytologic study of serous effusions might play a critical role in the rapid diagnosis of BL. In our case, the ascitic fluid study revealed the proliferation of monotonous non-cohesive cells with moderate basophilic vacuolated cytoplasm, non-cleaved nuclei, and multiple prominent nucleoli, similar to the results of a study by Haddad et al. on cytologic smears in Burkitt lymphoma [10,13].

Nevertheless, histopathologic examination of the neoplasm is essential to confirm the diagnosis. Microscopically, BL is characterized by a high proliferation fraction of monotonous, intermediate-sized cells with basophilic vacuolated cytoplasm, round nuclei, multiple nucleoli, and a high mitotic rate with the “classic starry sky pattern”. Furthermore, ancillary techniques are crucial to establish the final diagnosis. BL is derived from germinal and post-germinal center B-cells. Therefore, the neoplastic cells demonstrate positivity for B-cell markers including CD20, CD19, CD79a, and CD22, in addition to the positive expression of germinal center-associated antigens including CD10 and BCL6, with negative expression of TdT and BCL2 as in our case [[14], [15], [16]].

Regarding molecular genetics studies, all cases of BL harbor MYC translocation involving distinct locations. In approximately 80% of cases, this translocation involves the Myc gene on the long arm of chromosome 8 and the immunoglobulin heavy chain locus on chromosome 14. This translocation results in a high expression of c-Myc protein that regulates cell proliferation and apoptosis, which is responsible for the rapid tumor cell proliferation and subsequently, the high aggressiveness and the rapid doubling time (approximately 25 hours) of BL. Furthermore, sporadic BL cases are characterized by mutations in TCF3 gene and its negative regulator ID3 in approximately 70% of cases, in addition to a mutation in CCND3 in 38% of cases [[17], [18], [19]]. Nevertheless, molecular techniques are not available at our institution, and diagnosis was made based on detailed examinations in the presence of the aforementioned limitations.

Treatment of BL depends on brief-duration dose-intensive systemic chemotherapy regimens with multiple factors to be considered including the high proliferation rate of the tumor, age of the patient, and the risk of tumor lysis syndrome. In our case, the patient was treated based on the LMB protocol developed by The Societe Francaise d’Oncologie Pediatrique (SOFP) group which consists of alternating courses of Cyclophosphamide, Vincristine, Prednisone, Adriamycin, and Methotrexate. This protocol demonstrated a well-tolerance and improvement in survival rates among pediatric populations compared to adult groups. In our case, radiologic monitoring demonstrated a complete metabolic response to treatment and a marked improvement in our patient's status. On the other side, a large randomized trial performed by Ribrag et al. revealed an improvement in overall survival among adult and older populations upon adding Rituximab to the short intensive aforementioned regimen. Other treatment regimens include the Magrath protocol developed by Magrath et al. at the National Cancer Institute in the USA. This protocol consists of cyclophosphamide, vincristine, doxorubicin, high-dose Methotrexate (CODOX-M) alternating with ifosfamide, etoposide, and high-dose cytarabine (IVAC). In their original series, Magrath et al. reported similar prognosis rates among pediatric and adult populations. However, higher toxicity and myelosuppression-related deaths were reported. Nevertheless, the optimal chemotherapy regimen is still controversial due to the paucity of clinical trials [20,21].

## 4 Conclusions

In our case, we aimed to present a unique case that highlights several challenging features including the rare symptoms of gastrointestinal lymphomas, the major role of cytological study of ascites in the primary diagnosis, and the essential role of histological and immunohistochemical examinations in confirming the diagnosis in the absence of molecular techniques.

## Ethical approval

Research studies involving patients require ethical approval. Please state whether approval has been given, name the relevant ethics committee and the state the reference number for their judgement.

Not applicable. It's a case report.

## Sources of funding

All sources of funding should be declared as an acknowledgement at the end of the text. Authors should declare the role of study sponsors, if any, in the collection, analysis and interpretation of data; in the writing of the manuscript; and in the decision to submit the manuscript for publication. If the study sponsors had no such involvement, the authors should so state.

None declared. The research didn't receive any funding.

## Author contribution

Authors' contributions: SI: Drafted the manuscript. SA and IM: Collected the patient's data and participated in revising the article. AG: The pediatric oncologist: was in charge of the patient's treatment and status, provided the data and participated in drafting the manuscript. AD: Participated in the pathologic examination and revising the manuscript. ZA: The guarantor and supervisor, performed and confirmed the pathological diagnosis and critically revised the article. All authors have read and approved the manuscript.

## Registration of research studies

Not applicable. It's a case report.

## Guarantor

Dr. Zuheir Alshehabi.

## Consent

Written informed consent was obtained from the patient for publication of this case report and any accompanying images. A copy of the written consent is available for review by the Editor-in-Chief of this journal.

## Annals of medicine and surgery

The following information is required for submission. Please note that failure to respond to these questions/statements will mean your submission will be returned. If you have nothing to declare in any of these categories then this should be stated.

## Declaration of competing interest

All authors must disclose any financial and personal relationships with other people or organisations that could inappropriately influence (bias) their work. Examples of potential conflicts of interest include employment, consultancies, stock ownership, honoraria, paid expert testimony, patent applications/registrations, and grants or other funding.

None declared.
